# Metabolic cost of external work: a novel CPET parameter optimises characterisation of exercise performance in obese individuals

**DOI:** 10.1007/s00421-025-05929-5

**Published:** 2025-09-06

**Authors:** Jennifer J. Rayner, Rebecca R. Chamley, Robert Barker Davies, Oliver O’Sullivan, Peter Ladlow, Alex N. Bennett, Edward D. Nicol, Oliver J. Rider, David A. Holdsworth

**Affiliations:** 1 Division of Cardiovascular Medicine, Radcliffe Department of Medicine, Oxford Centre for Clinical Magnetic Resonance Research, University of Oxford, Oxford, UK; 2 Department of Cardiology, Oxford University Hospitals NHS Trust, John Radcliffe Hospital, Headley Way, Oxford, OX3 9DU UK; 3 Academic Department of Military Rehabilitation (ADMR), Defence Medical Rehabilitation Centre (DMRC), Stanford Hall, Loughborough, LE21 5QW UK; 4 Department for Health, University of Bath, Bath, UK; 5 Faculty of Medicine, National Heart and Lung Institute, Imperial College, London, UK; 6 Royal Brompton Hospital, London, UK

**Keywords:** Cardiorespiratory fitness, Cardiopulmonary exercise test, Obesity, Metabolic syndrome

## Abstract

**Purpose:**

Both obesity and cardiorespiratory fitness are crucial determinants of symptoms and prognosis. However, interpreting the gold-standard cardiopulmonary exercise test (CPET) is complicated by increasing body size and varying body composition. We hypothesised that the ‘metabolic cost of external work’ (or oxygen uptake (ml/min)/workload (Watts); V̇O_2_/W), a body weight-independent determinant of endurance capacity, would reflect metabolic health more accurately than V̇O_2_ alone.

**Methods:**

A test cohort of 160 fit individuals underwent anthropomorphic and metabolic assessment, maximal bicycle ergometer CPET, and six-minute walk test (6MWT). V̇O_2_/W was calculated at VT1 and peak. The performance of V̇O_2_/W was validated in 62 older, less fit individuals, undergoing the same protocol. 24 obese volunteers were assigned a weight loss intervention, and the impact on V̇O_2_/W examined.

**Results:**

In both test and validation cohort, V̇O_2_/W at VT1 and peak correlated with 6MWT distance, more strongly than standard CPET parameters. Including V̇O_2_/W improved the accuracy of predicting 6MWT distance. V̇O_2_/W correlated with BMI, insulin sensitivity and waist-to-hip ratio. Metabolic cost falls with weight loss, in parallel to metabolic and functional improvements, in contrast to other CPET parameters.

**Conclusion:**

Metabolic cost is strongly associated with functional capacity and metabolic health across a range of body weight and fitness, outperforming standard CPET metrics. It is a simple measure which may improve our assessment of the extent to which exertional symptoms are determined by metabolic factors in an individual, and thereby target the most appropriate intervention to those who will benefit most.

**Graphical Abstract:**

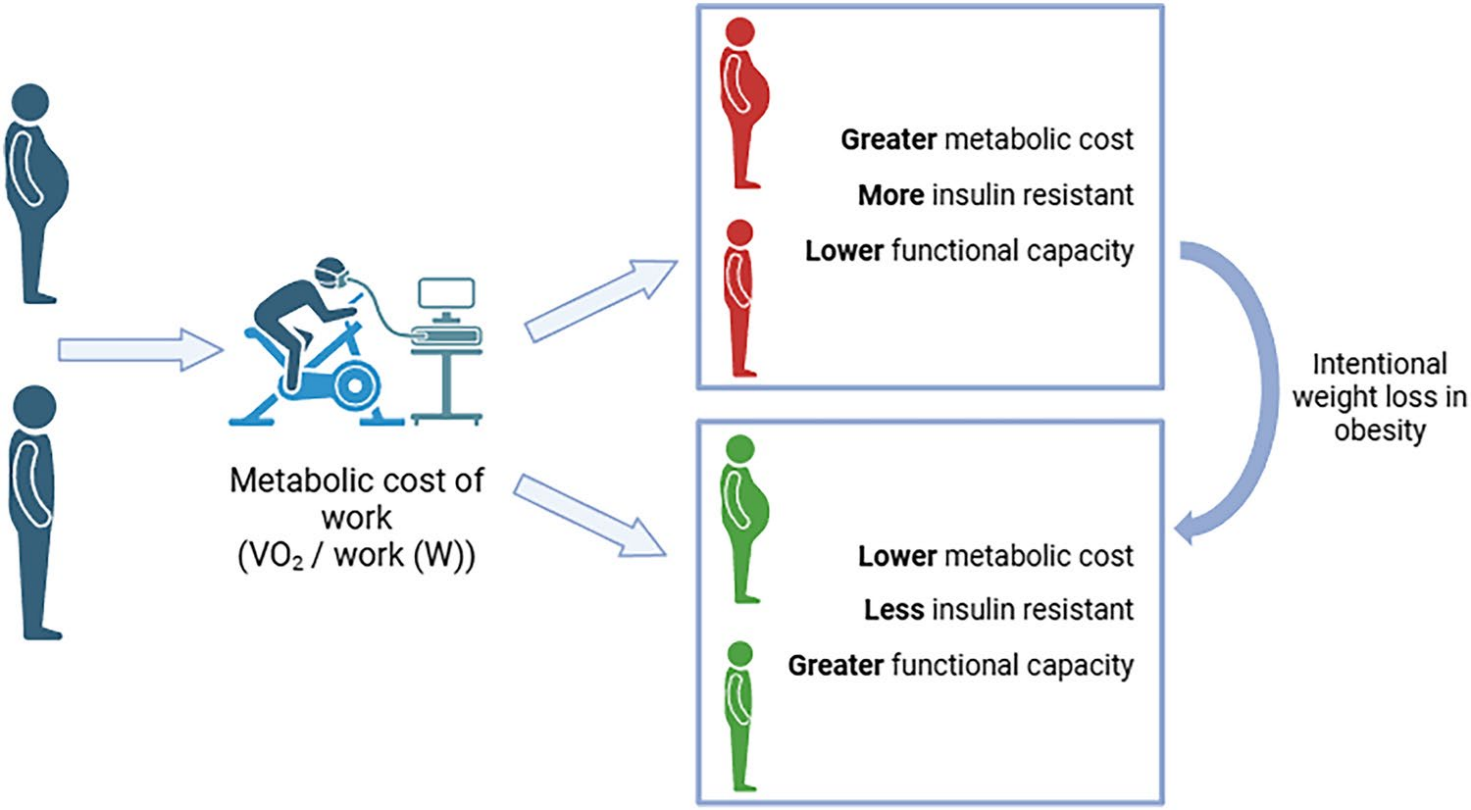

## Introduction

Increasing fat mass and obesity pose a significant challenge for the individual in terms of day-to-day functional capacity. On a population level, low volumes of physical activity are associated with greater risk of developing obesity, while being obese contributes to reduced activity levels (Pietilainen et al. 2008) – a vicious cycle of energy imbalance. However, the increased work of carrying a greater body weight exerts a gradual and progressive training effect which may offset the metabolic harm of obesity in some individuals. Certainly, for some, it is possible to have a high BMI and achieve high levels of cardiorespiratory fitness. In addition, there is extensive literature which would suggest that fitness is of equal and perhaps greater importance, than adiposity, in determining hard outcomes in a number of diseases (McAuley et al. 2012; Lavie et al. 2013; Hainer et al. 2009). Therefore, assessing fitness accurately is important across the spectrum of weight classes and body compositions.

Cardiopulmonary exercise testing is the gold-standard method for assessing whole-body fitness (Ross et al. 2016). Peak exercise capacity has been validated as a powerful predictor of all-cause mortality in many thousands of patients (Paffenbarger et al. 1986; Leon et al. 1987; Myers et al. 2002; Kokkinos et al. 2022). However, the standard parameters used become more difficult to interpret when comparing very different body sizes and compositions. Peak V̇O_2_ targets vary greatly across the range of sizes of adult humans. To try and allow for this, peak V̇O_2_ can be presented as a percentage of predicted values, based on the Wasserman weight-based algorithm (Wasserman et al. 2004), the product of weight (kg) and an age and sex-corrected constant. This algorithm provides a reliable and useful estimate of the peak V̇O_2_ in humans with a ‘normal’ body composition. However, it cannot differentiate between differing body compositions, and so ‘expects’ more from a heavier individual, no matter whether the extra mass is highly energy-demanding muscle, or relatively less metabolically active adipose tissue. It has been shown that correcting peak V̇O_2_ to lean mass outperforms standard methods in terms of prognosis (Osman et al. 2000; Lavie and Milani 2004).

The combination of increased body fat mass, decreased lean mass and reduced exercise/physical activity results in an altered profile of hormones and cytokines within the body, and adversely alters the tissue responses (skeletal muscle, liver and fat itself) to these hormones, and indeed to fuel substrates themselves (Clark 2012).

As body mass increases, the efficiency of physical work falls for two reasons. First, increasing energy expenditure is required to move the limbs and trunk *(internal work*) (Francescato et al. 1995). This accounts for the significantly higher oxygen consumption during unloaded pedalling for an obese individual on a cycle ergometer (Vaccari et al. 2019). Second, as a metabolic disease, obesity negatively impacts the efficiency of whole-body exercise performance in a number of ways. Reduced insulin sensitivity is associated with inefficient cardiac filling, impaired intramyocardial metabolism (Lopaschuk et al. 2007), reduced vascular compliance (Rider et al. 2016) and reduced efficiency of skeletal muscle energy metabolism (Mengeste et al. 2021). Overall, these effects result in less efficient transport and utilisation of oxygen to release the chemical energy stored in fuel substrates, and so reduced efficiency of contractile muscle function to power external work. Quantifying this inefficiency would enable more relevant comparison of fitness across a range of body composition and more accurately pinpoint the limiting factors responsible.

Cardiopulmonary exercise testing does include a parameter that links oxygen uptake and workload. The work rate slope is the gradient of the oxygen uptake (ml/min) plotted against workload (W) during the period of loaded exercise until the respiratory compensation point (RCP) (Wasserman and Whipp 1975). This parameter has been shown to vary with different rates of increase in external work (Hansen et al. 1988) and to differ above and below the anaerobic threshold (Lewalter et al. 1999). The true diagnostic benefit of the work rate slope is in demonstrating the absence of normal linear behaviour throughout the test, and the presence of abnormal early flattening that suggests myocardial ischaemia or other causes of stroke volume limitation such as heart failure (Belardinelli et al. 2003). By scrutinising the oxygen uptake per Watt at a specific comparable stage of a maximal exercise test, it may be possible to make inferences about metabolic fitness. This parameter offers a potential comparison of individuals of different size and body composition. Indeed, oxygen uptake corrected for work efficiency has been shown to capture cardiovascular changes in response to exercise training in obesity (Lavie and Milani 1999). The anaerobic threshold (most closely represented in CPET by the first ventilatory threshold: VT1) holds particular interest, given that this is a physiologically determined, and so comparable event in all humans, and also that the external work done at VT1 represents the amount of work that can be sustained for a prolonged period (Tanaka et al. 1984).

Our hypothesis was that the ratio of oxygen uptake (V̇O_2_) to external work (W) at VT1 would more accurately reflect metabolic health in a young, fit cohort of varying body composition than oxygen uptake alone. We then validated this parameter in an older, more obese cohort, in whom a subset was assigned a calorie-controlled weight loss intervention, to assess the dynamic impact of a reduction in body fat on exercise metabolism and work Finally, we examined the relative impact of body size and metabolic cost on overall cardiopulmonary fitness.

## Methods

The initial study cohort was recruited consecutively from a military population assessed as part of the Defence COVID recovery service (DCRS) between 2020 and 2022 (O'Sullivan et al. 2021), and those who had had severe COVID excluded. Ethical approval was received from the Ministry of Defence Research Ethics Committee (1061/MODREC/20). The validation cohort was retrospectively identified from a previous study of cardiorespiratory fitness in obesity between 2018 and 2020, with volunteers from a UK adult population. This was approved by the local research ethics committee (NRES Committee South Central 15/SC/0004), and all volunteers gave written consent for their anonymised data to be used in future studies.

### Anthropomorphic and biochemical assessment

Measurements were undertaken in the morning, fasted but with free hydration, with participants clothed but without shoes. Height, weight and body composition were measured using digital scales with bio-impedance analysis (InBody 770, InBody Co Ltd, South Korea). Body mass index (BMI) was calculated according to weight (kg) divided by height (m) squared. Waist to hip ratio measured according to the WHO consensus document 2008. Noninvasive blood pressure was measured according to standardised methods (average of three supine measurements following 5 min’ rest, with an automatic sphygmomanometer, Carescape V100, GE). Fasting venous blood was drawn and biomarkers were analysed either by the Oxford University Hospitals clinical biochemistry laboratory according to standardised protocols, or by commercially available ELISA kit (leptin; Sigma-Aldrich, St Louis, Missouris, USA). Fasting insulin resistance was represented by HOMA-IR ((glucose x insulin)/22.5).

### Cardiopulmonary exercise testing

All participants underwent maximal exercise testing on a cycle ergometer according to an individualised ramp protocol. Ramp gradient (W/min) was set to achieve 8–12 min of exercise. Continuous recording of respiratory gas exchange parameters was made during the test and recovery (Cortex Metalyzer 3b, CORTEX Biophysik GmbH, Leipzig, Germany). Participants were asked to exercise until volitional fatigue. Gas exchange data were acquired breath by breath and averaged over 20 s intervals. Peak V̇O_2_, heart rate, respiratory exchange ratio and VE/VCO_2_ slope were recorded.

### Six-minute walk test

All participants underwent a standardised walking test (Holland et al. 2014) along a 35 m lane for 6 min. The total distance achieved was recorded.

### Weight loss intervention

A prespecified subset of obese volunteers underwent dietary weight loss advice with telephone/email support from the study team. They adhered to a calorie-controlled (up to 1500 kcal), low glycaemic index diet for 387 ± 90 days. Volunteers were encouraged to maintain current activity levels during this time.

### Statistical analysis

Statistical analysis was performed using commercial software (SPSS 24, Chicago). All data are presented as mean ± standard deviation or median (interquartile range) where stated. Normality was assessed using a Shapiro–Wilk test. Parametric (paired and independent two-sided Student *t* tests) and nonparametric tests (independent samples Kruskal–Wallis test followed by Bonferroni correction for multiple tests; related samples Wilcoxon signed rank test) were used as appropriate. Significance across multiple groups was assess using one-way ANOVA, with Bonferroni correction. Pearson’s correlation and linear regression were used. *P* values of < 0.05 were considered as statistically significant.

## Results

### Test cohort

160 participants were included in the test cohort, of whom 134 were male (89%). This was a relatively young cohort, with a mean age of 38 ± 10 years, with higher-than-average levels of fitness when assessed with standard measures (peak V̇O_2_ 141 ± 36% predicted).

112 participants were normal weight or overweight (mean BMI 26 ± 2 kg/m^2^), and 48 were obese (mean BMI 33 ± 2 kg/m^2^; details in Table 1).

**Table 1:** Baseline parameters of test cohort participants divided according to body mass index

	Normal weight (*n* = 112)	Obese (*n* = 48)	*p*
Age (y)	37 ± 10	40 ± 9	0.072
Male sex (%)	90 (80)	44 (92)	0.076
Weight (kg)	83 ± 12	105 ± 13	< 0.001
BMI (kg/m^2^)	26 ± 2	33 ± 2	< 0.001
Waist-hip ratio	0.91 ± 0.08	0.98 ± 0.08	< 0.001
HbA1c	34 (31–36)	37 (33–39)	0.048*
Systolic blood pressure (mmHg)	119 ± 10	122 ± 11	0.109
Diastolic blood pressure (mmHg)	81 ± 9	83 ± 10	0.200
Heart rate (bpm)	83 ± 13	93 ± 15	< 0.001
Peak heart rate (% predicted)	109 ± 7	109 ± 9	0.735
Peak V̇O_2_ (ml/kg/min)	37 ± 7	31 ± 5	< 0.001
Absolute peak V̇O_2_ (ml/min)	3084 ± 700	3208 ± 593	0.283
Peak V̇O_2_ (% predicted)	144 ± 38	133 ± 31	0.108
Number greater than 100% predicted V̇O_2_ (%)	92 (82)	40 (83)	0.856
Peak V̇O_2_ (% predicted for lean body weight)	143 ± 25	130 ± 26	0.004
Work at VT1 (W)	82 ± 26	76 ± 19	0.149
Work at VT1 (W/kg)	1.0 ± 0.3	0.7 ± 0.2	< 0.001
Maximum work rate (W)	250 ± 55	244 ± 50	0.456
Maximum work rate (W/kg)	3.0 ± 0.5	2.3 ± 0.5	< 0.001
Peak RER	1.19 ± 0.05	1.15 ± 0.05	< 0.001
VE/VCO_2_ slope	28 ± 5	29 ± 5	0.075
Six-minute walk test (m)	625 ± 96	594 ± 78	0.070
Metabolic cost (ml/min/W) at VT1	12.4 ± 1.3	13.2 ± 1.2	< 0.001
Metabolic cost (ml/min/W) at peak	15.5 ± 2.4	17.9 ± 3.3	< 0.001

^*^Nonparametric data presented as median (interquartile range); differences assessed by MWU test

### Impact of obesity on baseline parameters

There were no statistically significant differences between the obese group (Ob) and the normal/overweight group (Ln) in terms of age (40 ± 9 years compared to 37 ± 10 years; *p* = 0.072), or sex (92% male vs 80%; *p* = 0.076). The Ob group was more insulin resistant (HbA1c 37 (33–39) mmol/mol vs 34 (31–36) mmol/l; *p* = 0.048), and had higher resting heart rate (93 ± 15bpm vs 83 ± 13bpm; *p* < 0.001). There was evidence of more central distribution of adipose tissue with higher waist-to-hip ratio in the Ob group (0.98 ± 0.08 vs 0.91 ± 0.08; *p* < 0.001).

### Impact of obesity on cardiorespiratory fitness

Using six-minute walk test as a measurement of submaximal exercise capacity, there were no differences between Ln and Ob groups (625 ± 96m compared to 594 ± 78 m, *p* = 0.070).

**Fig. 1:**
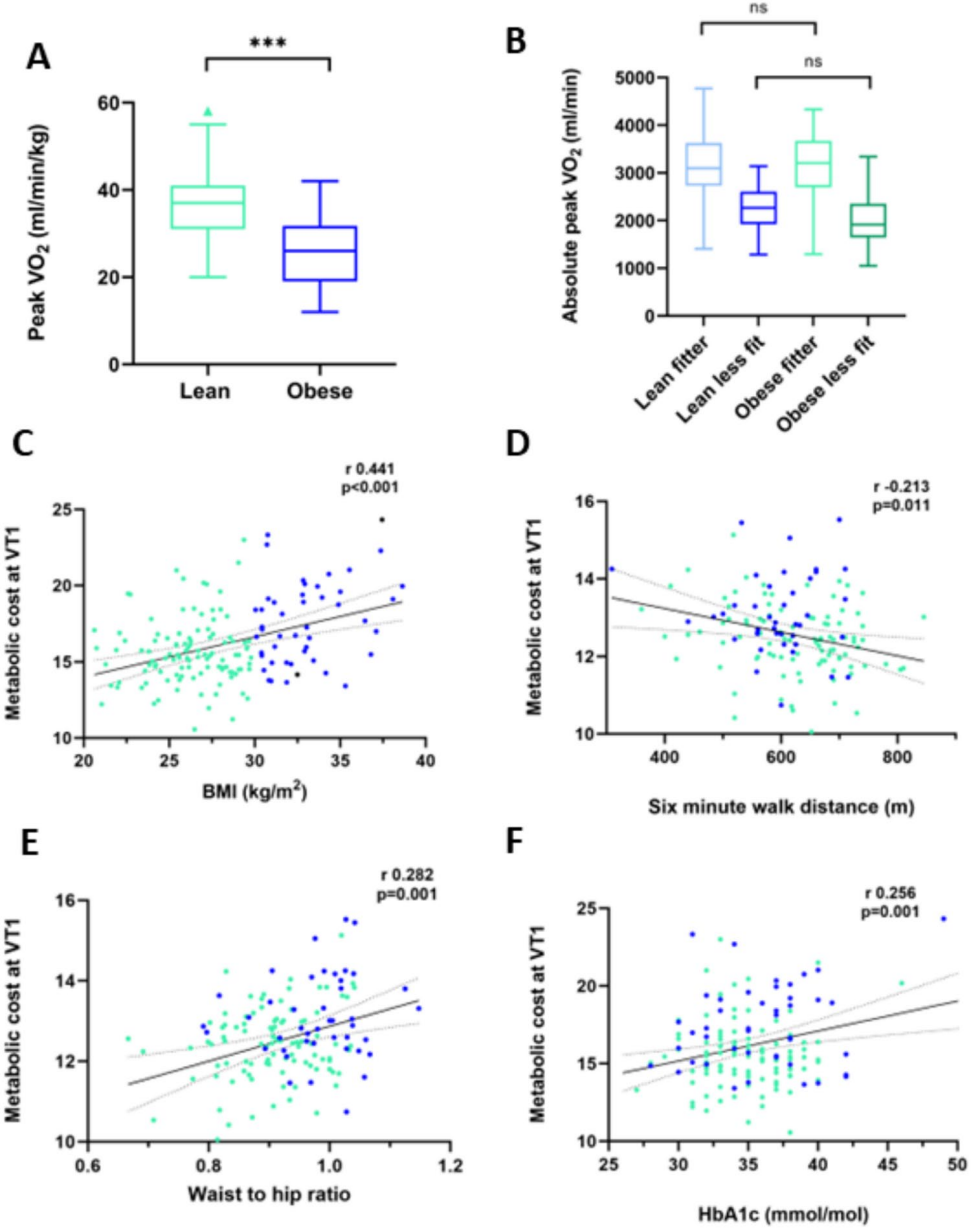
Using standardised assessments of cardiorespiratory fitness is disproportionately affected by body weight (**A**) and does not discriminate between fitter individuals of differing weight (**B**). Measuring the ‘metabolic cost’ (ie oxygen uptake divided by external work) correlates with BMI (**C**) but also with functional capacity (**D**), and parameters associated with metabolic fitness (**E and F**). Results displayed from test cohort

CPET testing was maximal across the cohort, with peak RER 1.15 ± 0.05 in the Ob group, and 1.19 ± 0.05 in the Ln group (*p* < 0.001), and peak heart rate 109 ± 9% predicted in the Ob group and 109 ± 7% predicted in the Ln group (*p* = 0.735). Despite no difference in peak V̇O_2_ between Ob and Ln groups (3208 ± 593 ml/min vs 3084 ± 700 ml/min, *p* = 0.283), when this value was then indexed to body weight, the Ob group achieved a lower value (31 ± 5 ml/min/kg vs 37 ± 7 ml/min/kg; *p* < 0.001; Fig. 1A).

However, peak V̇O_2_ in terms of percentage predicted was both supranormal and not different between groups (Ob 133 ± 38% vs Ln 144 ± 38%, *p* = 0.108). If the cohorts were separated into more (> 100% predicted peak V̇O_2_ achieved) and less fit (< 100% predicted peak V̇O_2_ achieved), the absolute V̇O_2_ was no different between lean and obese ‘less fit’ groups, and lean and obese ‘more fit’ groups (Fig. 1B).

### Using ‘metabolic cost’ of external work

The ‘metabolic cost’ of external work was calculated as absolute V̇O_2_ (ml/min)/work rate (W) and was examined both at VT1 and peak. These values correlated negatively with six-minute walk test distance (*r *= − 0.213, *p* = 0.011; Fig. 1D). In other words, the greater the metabolic cost, the lower the distance achieved. While the calculated values for V̇O_2_/WR at both VT1 and at peak correlated with six-minute walk test distance, the value for overall work rate slope (V̇O_2_/W) did not (r − 0.103, *p* = 0.228). In addition, metabolic cost correlated with both BMI (Fig. 1C) and with factors indicating overall metabolic health (waist-to-hip ratio, Fig. 1E; insulin resistance Fig. 1F). Again, there was no significant relationship between work rate slope and insulin resistance (r 0.046, *p* = 0.551) or waist-to-hip ratio (r 0.156, *p* = 0.151).

On multiple regression analysis, with six-minute walk test distance as the dependent variable, metabolic cost was more closely associated than body weight (β − 0.241, *p* = 0.006 compared to β 0.002, *p* = 0.980), age (age β 0.182, *p* = 0.033) or peak V̇O_2_ (β 0.216, *p* = 0.011). In addition , on stepwise regression with six-minute walk test as the dependent variable, and including age and peak V̇O_2_, adding metabolic cost to the model increases the adjusted R^2^ from 0.044 to 0.102, indicating that this novel parameter improves the fit of the model.

### Validation cohort

To assess whether the novel metabolic cost parameter held the same associations, and improved the accuracy of predicting submaximal exercise performance, measured by six-minute walk test in a different population, a community-recruited cohort of 62 individuals was used for validation (comparison with test cohort in Table 2).

**Table 2:** Baseline parameters of test vs validation cohort participants

	Test cohort (*n* = 160)	Validation cohort (*n* = 62)	*p*
Age (y)	38 ± 10	48 ± 15	< 0.001
Male sex (%)	134 (84)	16 (26)	< 0.001
Weight (kg)	89 ± 16	93 ± 22	0.251
BMI (kg/m^2^)	28 ± 4	33 ± 7	< 0.001
Waist-hip ratio	0.93 ± 0.09	0.91 ± 0.10	0.159
Systolic blood pressure (mmHg)	120 ± 10	134 ± 18	< 0.001
Diastolic blood pressure (mmHg)	81 ± 9	84 ± 12	0.141
Heart rate (bpm)	85 ± 14	83 ± 14	0.488
Peak heart rate (% predicted)	108 ± 8	87 ± 20	< 0.001
Peak V̇O_2_ (ml/kg/min)	36 ± 7	23 ± 8	< 0.001
Absolute peak V̇O_2_ (ml/min)	3121 ± 670	2009 ± 600	< 0.001
Peak V̇O_2_ (% predicted)	141 ± 36	93 ± 22	< 0.001
Number greater than 100% predicted V̇O_2_ (%)	132 (83)	17 (27)	< 0.001
Maximum work rate (W)	248 ± 54	158 ± 48	< 0.001
Maximum work rate (W/kg)	2.8 ± 0.6	1.8 ± 0.7	< 0.001
Peak RER	1.18 ± 0.05	1.14 ± 0.06	< 0.001
VE/V̇CO_2_ slope	28 ± 5	26 ± 4	0.004
Six-minute walk test (m)	616 ± 92	583 ± 66	0.008
Metabolic cost (ml/min/W) at VT1	12.6 ± 1.3	12.8 ± 1.2	0.157
Metabolic cost (ml/min/W) at peak	16.2 ± 2.9	18.0 ± 4.8	0.006

The validation cohort was older (48 ± 15 y vs 38 ± 10, *p* < 0.001), predominantly female (26% men compared to 84% of the test cohort, *p* < 0.001), and had higher BMI (33 ± 7 kg/m^2^ vs 28 ± 4 kg/m^2^, *p* < 0.001). Although CPET tests were maximal in both groups (peak RER in both cohorts > 1.1), peak heart rate achieved was lower in the validation cohort (87 ± 20% predicted peak, as compared to 108 ± 8%, *p* < 0.001). Although the peak V̇O_2_ reached in the validation cohort was within normal clinical limits (93 ± 22% predicted), all measured parameters were lower than the test cohort (see Table 2), indicating this was a less fit cohort, more reflective of the general population.

Despite the cohort having different characteristics, metabolic cost continued to correlate with six-minute walk test distance (r − 0.326, *p* = 0.025), with BMI (r 0.632, *p* < 0.001) and insulin resistance (r 0.449, *p* = 0.002). On stepwise multiple regression with six-minute walk test as the dependent variable, including age and peak V̇O_2_ as independent variables, including metabolic cost slightly improves the fit (R^2^ from 0.312 to 0.319).

### Performance according to efficiency and BMI

To understand more about the relationship between BMI and the metabolic cost parameter, both test and validation cohorts were combined, and then divided according to BMI characteristics as above. Metabolic cost was greater in the Ob group at both VT1 (18 ± 4 vs 15 ± 2, *p* < 0.001) and peak (13 ± 1 vs 12 ± 1, *p* < 0.001); however, there was significant overlap indicating that some obese individuals had lower metabolic cost of external work (were ‘more efficient’) and some lean individuals had higher metabolic cost (were ‘less efficient’).

The two body composition groups were subdivided around the mean value for metabolic cost at VT1 (mean for whole cohort 16.2), resulting in a division into lean efficient (Ln_e_; *n* = 87), lean inefficient (Ln_i_; *n* = 24), obese efficient (Ob_e_; *n* = 18) and obese inefficient (Ob_i_; *n* = 30). There were no statistically significant differences in participant characteristics, markers of inflammation or insulin resistance, resting or exertional CPET parameters between Ln_e_ and Ln_i_ groups (Table 3), with the exception of peak diastolic blood pressure being lower in Ln_e_ (76 ± 16 mmHg compared to 84 ± 12 mmHg in Ln_i_, *p* = 0.015).

**Table 3:** The cohort divided according to BMI classification, and high vs low metabolic cost at peak (Ln_e_ lean efficient; Ln_i_ lean inefficient; Ob_e_ obese efficient; Ob_i_ obese inefficient)

	Ln_e_ (*n* = 75)	Ln_i_ (*n* = 48)	Ob_e_ (*n* = 28)	Ob_i_ (*n* = 54)
Male sex (%)	57 (76)	37 (77)	26 (93)	46 (85)
Age (y)	37 ± 10	42 ± 14	44 ± 13	44 ± 13*
BMI (kg/m^2^)	26 ± 2	26 ± 3	34 ± 3*‡	34 ± 4*‡
Waist/hip ratio	0.90 ± 0.09	0.91 ± 0.07	0.97 ± 0.08*‡	0.96 ± 0.09*
CRP**	0.9 (0.6–1.5)	0.9 (0.6–1.5)	1.7 (0.8–2.4)	1.9 (0.8–2.8) *‡
HbA1c**	35 (33–37)	34 (33–36)	36 (32–39)	37 (33–39)
HOMA-IR**	1.5 (1.1–1.8)	2.9 (1.6–4.3)	2.8 (1.2–4.7)	3.2 (1.9–5.9)*
HR at rest (bpm)	83 ± 12	82 ± 14	89 ± 14	90 ± 16*‡
Peak HR (% max)	106 ± 10	107 ± 10	103 ± 13	100 ± 14*‡
SBP at rest (mmHg)	120 ± 13	124 ± 12	130 ± 17*	127 ± 14*
DBP at rest (mmHg)	82 ± 9	82 ± 11	81 ± 10	84 ± 11
SBP at peak (mmHg)	165 ± 19	169 ± 20	175 ± 24	179 ± 22*
DBP at peak (mmHg)	76 ± 16	84 ± 12*	88 ± 15*	84 ± 15*
Six-minute walk test (m)	635 ± 86	606 ± 96	597 ± 73	575 ± 77*
V̇O_2_/W at VT1	15.0 ± 2.2	16.1 ± 2.7	17.1 ± 4.1*	19.0 ± 4.1*‡
V̇O_2_/W at peak	11.7 ± 1.1	13.4 ± 0.5*	12.0 ± 0.6‡	13.7 ± 0.9*†
Max work (W)	246 ± 62	233 ± 61	223 ± 80	192 ± 57*‡
W/kg	3.1 ± 0.6	2.8 ± 0.6	2.1 ± 0.6*‡	1.9 ± 0.5*‡
V̇O_2_ peak (ml/kg/min)	36 ± 7	37 ± 8	25 ± 9*‡	26 ± 7*‡
V̇O_2_ peak (% predicted)	141 ± 38	131 ± 42	117 ± 37*	111 ± 34*
O_2_ pulse (% predicted)	167 ± 47	178 ± 53	118 ± 38*‡	115 ± 29*‡
RER peak	1.18 ± 0.06	1.17 ± 0.06	1.15 ± 0.05*	1.14 ± 0.06*
VE/V̇CO_2_ slope	28 ± 4	28 ± 5	29 ± 4	28 ± 5

^*^Indicates significant difference to Ln_e_

^**^Kruskal Wallis test for nonparametric data

^‡^Significant difference to Ln_i_

^†^Indicates significant difference to Ob_e_ (Ob_i_ only)

When comparing efficient vs inefficient in the obese cohorts, there were no statistical differences in age, BMI, or standard CPET parameters between these groups. However, CRP was greater in the obese inefficient group (Ob_i_ 3.2 (1.9–5.9); Ob_e_ 2.8 (1.2–4.7); *p* = 0.039).

There were interesting findings when both obese groups were compared to the nonobese efficient group. Both CRP and HOMA-IR were higher in the Ob_i_ cohort but not Ob_e_ (CRP Ln_e_ 0.9 (0.6–1.5); Ob_e_ 2.8 (1.2–4.7) *p* = 0.157 compared to Ln_e_; Ob_i_ 3.2 (1.9–5.9) *p* = 0.015; HOMA-IR Ln_e_ 1.5 (1.1–1.8); Ob_e_ 2.8 (1.2–4.7) *p* = 0.303 compared to Ln_e_; Ob_i_ 3.2 (1.9–5.9) *p* = 0.010). In a similar pattern, resting heart rate was higher in the Ob_i_ group than in Ln_e_ (92 ± 16 bpm vs 83 ± 13 bpm, *p* = 0.018) but not in Ob_e_ (86 ± 15 bpm, *p* = 0.221), and six-minute walk test distance was lower in Ob_i_ but not Ob_e_ (Ln_e_ 633 ± 87m; Ob_i_ 572 ± 83m, *p* = 0.001; Ob_e_ 598 ± 61 m, *p* = 0.344 vs Ln_e_).

**Fig. 2:**
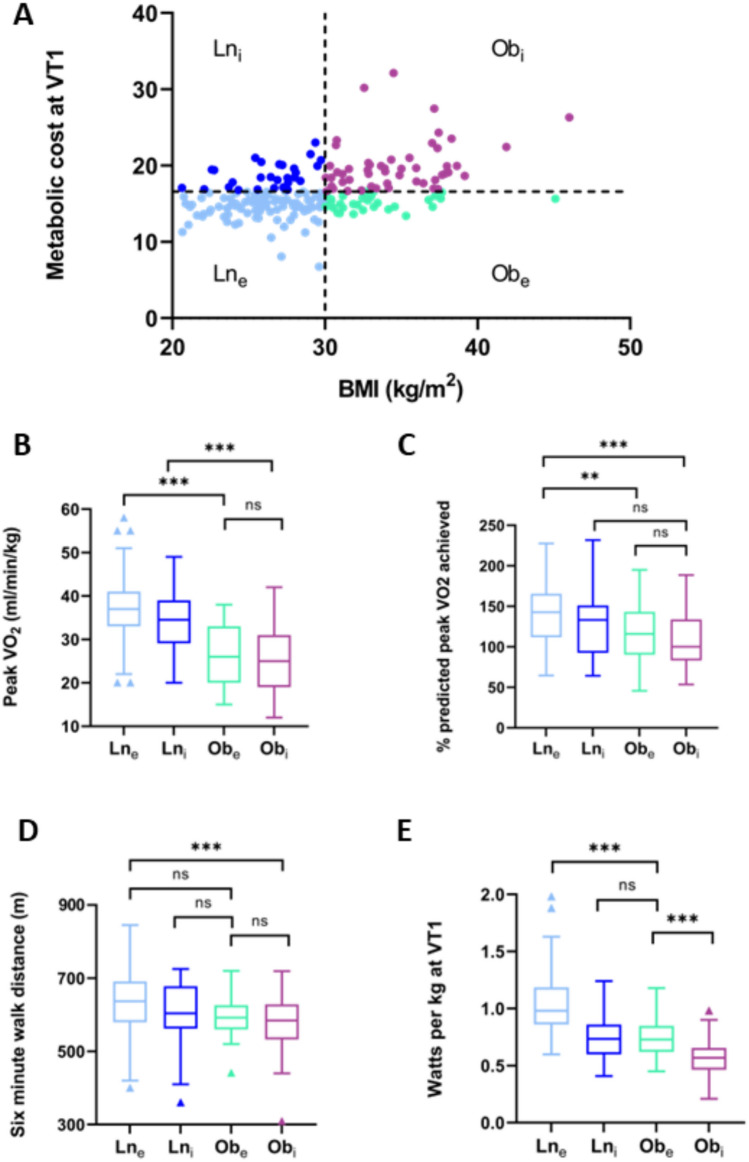
Dividing the cohort into lean and obese, and low vs high metabolic cost at VT1 (around the mean) demonstrates that peak V̇O_2_ is still dominated by body weight (**B**), but that lean and obese inefficient groups are no different in terms of % predicted peak V̇O_2_ (**C**), and that lean inefficient and obese efficient are equivalent for six-minute walk test distance (**D**) and for power output indexed to body weight (**E**)

While weight-dependent measures such as indexed peak V̇O_2_ still demonstrate significant differences between obese and lean groups (Fig. 2B), parameters including % predicted V̇O_2_ showed no statistical difference between lean and obese inefficient groups (Ln_i_ 131 ± 42%, Ob_i_ 117 ± 37%, *p* = 1.0).

### The impact of weight loss on efficiency of power output

A subset of 24 obese volunteers adhered to a calorie-controlled weight loss programme, over a period of 387 ± 90 days. Of these, 19 lost more than 5% total body weight (− 13 ± 5%), whereas the remaining 5 lost less than 5% body weight (+ 0.5 ± 6%).

**Fig. 3:**
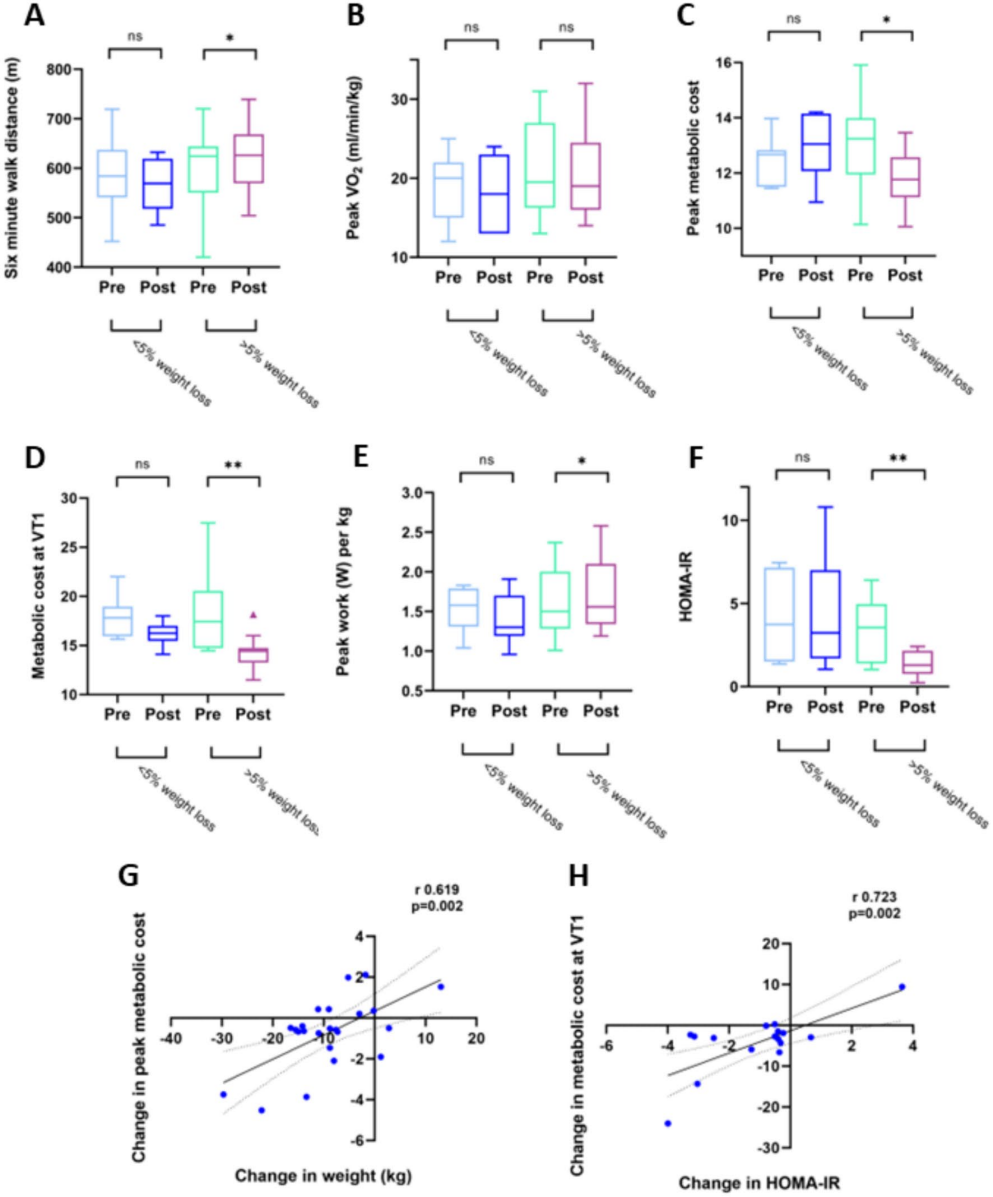
The improved exercise tolerance achieved by intentional weight loss of greater than 5% body weight leads to greater six-minute walk test distance (**A**), but this is not reflected in peak V̇O_2_ indexed to body weight (**B**). Weight loss results in lower oxygen consumption per watt of power output at peak (**C**) and anaerobic threshold (**D**), as well as greater work per kg body weight (**E**) and improved insulin resistance (**F**). Decreased body weight (**G**) and improved insulin sensitivity (**H**) are both correlated with improvement in metabolic cost

Weight loss was associated with improvements in resting cardiometabolic status (HOMA-IR 3.6 (1.4–5.0) to 1.3 (0.8–2.2), *p* = 0.002 (Wilcoxon Signed Ranks test); (Fig. 3); diastolic blood pressure 87 ± 12 mmHg to 82 ± 10 mmHg, *p* = 0.042; Table 4). Six-minute walk test distance improved significantly (593 ± 86 m to 625 ± 71 m, *p* = 0.031). Despite this, there was no significant change in indexed V̇O_2_ (21 ± 6 ml/kg/min to 21 ± 5 ml/kg/min, *p* = 0.787) or % predicted V̇O_2_ (89 ± 13% to 90 ± 14%, *p* = 0.420). This was due to balanced, parallel reductions in weight and absolute V̇O_2_ (2144 ml/min to 1833 ml/min, *p* < 0.001) due to reduced overall body size.

**Table 4:** The impact of intentional weight loss in obesity

	< 5% weight loss *n* = 5		> 5% weight loss *n* = 19	
	Pre	Post	Pre	Post
BMI (kg/m^2^)	36 ± 5	36 ± 6	36 ± 6	31 ± 7***
Weight (kg)	97 ± 19	99 ± 23	102 ± 18	89 ± 16***
HOMA-IR	4.0 (2.0–7.2)	2.9 (1.4–7.0)	3.6 (1.4–5.0)	1.3 (0.8–2.2)**
HR at rest	83 ± 13	91 ± 9	84 ± 16	80 ± 8
Peak HR (% max)	89 ± 6	88 ± 10	89 ± 12	96 ± 9*
SBP at rest (mmHg)	141 ± 18	147 ± 18	138 ± 25	127 ± 8
DBP at rest (mmHg)	85 ± 14	86 ± 15	87 ± 12	82 ± 10*
SBP at peak (mmHg)	182 ± 37	194 ± 37	185 ± 25	177 ± 21
DBP at peak (mmHg)	91 ± 13	90 ± 1.4	92 ± 8	87 ± 9*
Six-minute walk test (m)	582 ± 83	563 ± 52	593 ± 86	625 ± 71*
Max work (W)	144 ± 34	134 ± 33	165 ± 53	156 ± 42
Peak W/kg	1.5 ± 0.3	1.4 ± 0.3	1.6 ± 0.4	1.8 ± 0.5*
V̇O_2_ peak (ml/kg/min)	19 ± 5	18 ± 4	21 ± 6	21 ± 5
V̇O_2_ peak (% predicted)	87 ± 32	90 ± 32	89 ± 13	90 ± 14
O_2_ pulse (% predicted)	105 ± 34	107 ± 31	96 ± 18	95 ± 18
RER peak	1.18 ± 0.05	1.11 ± 0.04**	1.13 ± 0.06	1.14 ± 0.06
VE/V̇CO_2_ slope	26 ± 5	26 ± 4	26 ± 4	28 ± 5**
Metabolic cost (V̇O_2_/W) at peak	12.5 ± 0.9	13.0 ± 1.2	13.1 ± 1.4	11.8 ± 1.0**
Metabolic cost (V̇O_2_/W) at VT1	19.4 ± 5.7	17.0 ± 4.8	19.5 ± 6.4	14.3 ± 1.4**

^*^Level of significance vs ‘pre’ in same group (*p < 0.05; **p < 0.01; ***p < 0.001)

In the group that lost weight, there was a 12.5% increase in peak work per kg (1.6 W/kg to 1.8 W/kg, *p* = 0.016). Looking at the metabolic cost efficiency parameter, V̇O_2_/work fell significantly both at peak (by 15%, 13.1 ± 1.4–11.9 ± 1.0, *p* = 0.005) and at VT1 (by 27% 19.5 ± 6.4–14.3 ± 1.4, p = 0.004). In both cases, this was due to lower V̇O_2_ with no reduction in peak work.

Across the whole weight intervention cohort, change in weight correlated significantly with change in metabolic cost at peak (r 0.619, p = 0.002), and change in metabolic cost correlated with change in HOMA-IR (r 0.723, p = 0.002).

## Discussion

Increased body weight and fat proportion can contribute to exertional limitation, and are implicated in impairment of cardiorespiratory fitness. The standard parameters of cardiopulmonary exercise testing are appropriately determined according to body size; however, the reference data are based on a historic population with healthy body composition. Interpreting results is challenging in a population with excess adipose tissue. Obesity can result in decreased efficiency both mechanically and metabolically. Normal peak oxygen uptake targets are harder to attain in obese individuals, because these targets are based on the assumption that the body is composed of a healthy proportion of energy-demanding skeletal muscle. We tested a body weight-independent measure of cardiorespiratory efficiency, using a cohort of fit individuals across a wide range of body sizes. We found that measuring the oxygen uptake normalised to work done (V̇O_2_/W), the ‘metabolic cost’ at both VT1 and peak correlated well with BMI but also with measures of metabolic health, enabling differentiation of the cohort not only according to body size but also potentially metabolic efficiency. Metabolic cost highlighted the beneficial effect of weight loss, which neither peak workload nor peak oxygen uptake could detect. This novel weight-independent parameter based on a standard CPET examination increases the accuracy of assessing both functional capacity, and beneficial impact of weight loss, across the range of body sizes.

### The problem with CPET interpretation in obesity

To appreciate the complexity of interpreting CPET parameters in an obese population, we need to consider the basic test parameters. The Fick principle defines oxygen uptake (V̇O_2_) as the product of cardiac output (stroke volume x heart rate) and the difference in arterial and venous oxygen content:

$${VO}_{2}=\left(SVxHR\right) x ({CaO}_{2}-{CvO}_{2})$$

This represents an integrative measure of whole-body capacity to take up, transport and use oxygen to permit oxidative metabolism. During exercise, energy metabolism is coupled to the development of external mechanical work. It is of limited utility to compare the absolute peak oxygen uptake (L/min) of a healthy, lean 90 kg man and a healthy, lean 50 kg woman. A more helpful comparison can be achieved by normalising to body weight (ml/min/kg). However, there are also inherent sex-based differences in peak oxygen uptake, and the peak oxygen uptake also falls throughout adult life.

The interpretation of peak V̇O_2_ can therefore be enhanced by comparing the values attained against a control population of the same sex and age. However, the Equations. (Wasserman et al. 2004) which are used to calculate the predicted values assume a ‘normal’ proportion of metabolically active tissue. Obese individuals are therefore likely to fall short of predicted peak V̇O_2_ values, as the calculations which predict the target overestimate the proportion of metabolically active tissue. Even when obese individuals *do* achieve peak oxygen uptake in the normal range, this can offer false reassurance as the peak external work may be significantly reduced and the peak oxygen uptake elevated by significant inefficiencies of oxygen utilisation. The relationship between oxygen uptake and ‘useful work done’ reflects metabolic efficiency.

The corollary of this observation is highlighted when interpreting the impact of intentional body weight reduction in an obese individual. Absolute V̇O_2_ following significant body weight reduction is reduced, as the metabolically active mass is smaller. This does not mean that the individual is less fit. When the absolute V̇O_2_ value is normalised to the reduced body weight, the cardiorespiratory fitness (ml/min/kg) may be unchanged. However, if the peak work capacity falls less than the oxygen uptake this reflects increased efficiency, particularly for load-bearing activity. As a result, objective exercise performance (e.g. 6 min walk) and quality of life, may improve significantly.

Not only are typical CPET measures inadequate to permit baseline characterisation of obese individuals, but they do not permit straightforward comparison of cardiorespiratory fitness across a range of body sizes. A number of studies have found that physical activity and fitness are of greater importance than BMI in determining disease prognosis (Hainer et al. 2009; McAuley and Beavers 2014; Lavie et al. 2003). It is clearly desirable to find a measure of fitness that operates across the spectrum of body weight and composition. Such a metric may also be anticipated to provide a more reliable index of metabolic health and even of disease prognosis.

### Efficiency of oxygen utilisation is associated with insulin resistance

We propose a measure, *metabolic cost*, which incorporates not only oxygen uptake, but the efficiency of oxygen utilisation for skeletal muscle contraction and the development of external work. This parameter captures a number of factors, including the internal work required to move larger limbs and trunk, the effective mass of skeletal muscle and the metabolic health of the cardiac and skeletal muscle. This novel parameter is readily attainable from standard CPET data and has the potential to improve risk stratification in established disease through more accurate assessment of cardiopulmonary fitness, as well as identifying metabolic components for reduced exercise tolerance.

We found it to be well correlated with six-minute walk test distance and superior to body weight, age or, importantly, work rate slope in predicting distance achieved. When included in a model with age, weight and peak V̇O_2_, metabolic cost increased the accuracy of six-minute walk test distance prediction in both test and validation cohorts. Whilst metabolic cost is correlated with BMI, there is a wide range of exercise efficiency at any given BMI/body composition. Metabolic cost identifies efficiency independent of body composition and is associated with exercise performance. On a number of measures including metabolic (HOMA-IR), haemodynamic (resting heart rate) and functional (six-minute walk distance), there was no difference between the obese efficient group and lean groups, suggesting that metabolic cost is able to discriminate a heavier but functionally and metabolically fitter group.

We found that the metabolic cost of external work was associated with markers of metabolic health, in particular waist-to-hip ratio and insulin sensitivity. The greater the waist-to-hip ratio, or degree of insulin resistance, the greater the metabolic cost. This suggests a link with glucose metabolism, which is associated with impaired oxidative metabolism at several levels. Firstly, inability of the insulin-resistant myocardium to access glycolytic pathways leads to an increased energy cost of both contraction and relaxation (Peterson et al. 2004), particularly unmasked during stress (Rayner et al. 2020). Secondly, insulin resistance is associated with vascular dysfunction (Rider et al. 2016), which may impair muscle perfusion during exercise. Finally, skeletal muscle performance is degraded, with a change in fibre type from slow to fast twitch (Tallis et al. 2018), alteration in intracellular signalling cascades (Steinberg et al. 2006), and impaired intracellular energy metabolism (Klepochova et al. 2017; Valkovic et al. 2016). Our data suggest that even in a young and fit population, we can use this novel parameter to identify functional impairment associated with insulin resistance and tailor appropriate interventions accordingly.

### Metabolic cost of work falls with weight loss

In the weight loss cohort, we demonstrated that despite impressive body fat reduction, associated cardiometabolic improvements and increased distance achieved during six-minute walk testing, there was no measurable change in standard CPET parameters corresponding to the benefits yielded. In contrast, the metabolic cost of external work fell at both VT1 and peak. At VT1, this 26% improvement was driven by greater workload with no increase in oxygen uptake. At peak, the 10% improvement in metabolic cost was driven by a reduced oxygen requirement despite unchanged peak workload.

This demonstrates the beneficial effects of body fat reduction, with maintained power output despite a lower oxygen uptake, and supports the hypothesis of improved organ performance at multiple sites as a result of enhanced insulin sensitivity and other beneficial improvements to tissue responses and the hormonal milieu. Further support of this link between metabolic and functional improvement is provided by the correlation between whole body insulin resistance (primarily driven by skeletal muscle insulin sensitivity) and reduction in metabolic cost.

### Limitations

In the absence of more comprehensive assessment of either whole-body or organ-level insulin sensitivity, the conclusions drawn from this study are naturally exploratory. However, the findings are both valid in a ‘real-world’ population, and impacted by body weight modifying interventions. It would be interesting to see how a training intervention impacted metabolic cost, as there is likely to be an impact from deconditioning and physical exercise history. Unfortunately training and weight history were not available amongst study participants, which would be likely to cause some variability in exercise responses. The weight loss intervention itself was not prescriptive in terms of macro- or micronutrient components, and there may be impact of nutritional changes which are not captured.

## Conclusion

Understanding and accurately assessing cardiorespiratory fitness is particularly important in increasing body sizes, given the synergistic impact of obesity and low fitness on prognosis in a wide range of conditions. The interpretation of traditional CPET parameters is largely biased towards a lean body composition. Where there is an excess of metabolically less active adipose tissue, target oxygen uptake is more difficult to achieve. Inefficiencies resulting from obesity are reflected in reduced oxygen uptake, but also in a disproportionate reduction in external work. We use a novel parameter, the ‘metabolic cost of external work’, to improve the description of cardiopulmonary fitness and efficiency across a range of body sizes.

Metabolic cost improved the accuracy of interpreting CPET data and was more closely associated with six-minute walk test distance than other traditional parameters. It was associated with BMI, but importantly also with other indicators of metabolic health. Metabolic cost improved with intentional body fat reduction, more sensitively than any other standard CPET parameter.

Improving the assessment of cardiorespiratory fitness in obesity will enable greater understanding of the relative contribution of body weight and metabolic health to exertional symptoms, and therefore to target specific lifestyle interventions to those who will benefit most.

## Data Availability

All data are available for review at reasonable request from the corresponding author.
